# An investigation of the antibacterial ability and cytotoxicity of a novel cu-bearing 317L stainless steel

**DOI:** 10.1038/srep29244

**Published:** 2016-07-07

**Authors:** Da Sun, Dake Xu, Chunguang Yang, M. Babar Shahzad, Ziqing Sun, Jin Xia, Jinlong Zhao, Tingyue Gu, Ke Yang, Guixue Wang

**Affiliations:** 1 Key Laboratory of Biorheological Science and Technology, College of Bioengineering, Chongqing University, Chongqing 400044, China; 2 Institute of Metal Research, Chinese Academy of Sciences, 72 Wenhua Road, Shenyang 110016, China; 3 College of Chemistry, Liaoning University, Shenyang 110036, China; 4 Department of Chemical and Biomolecular Engineering, Institute for Corrosion and Multiphase Technology, Ohio University, Athens, OH 45701, USA

## Abstract

In order to solve the challenging problem of microbial infections caused by microorganisms on medical implants, it is imperative to develop novel antimicrobial biomaterials. This work demonstrated that 317L-Cu stainless steel (SS), created by adding copper through a solution and aging heat treatment process, exhibited good antibacterial properties against *staphylococcus aureus*, achieving 2 log reduction of planktonic cells after 5 days of incubation. In this study, the antibacterial test was performed using the plate count method, the fluorescence cell staining method and the quantitative polymerase chain reaction (qPCR) method. It is well known that a high concentration of copper ion can lead to cytotoxicity. This work explored the cytotoxicity of 317L-Cu SS through real-time cell analysis (RTCA). Experimental results demonstrated that the 317L-Cu SS possessed a satisfactory antibacterial ability against *S. aureus*, and the antibacterial rate based on the reduction of sessile cell count reached 98.3% after 24-hour treatment. The bacterial adhesion and the biofilm thickness were considerably reduced by the 317L-Cu SS. The results of RTCA suggested that 317L-Cu SS did not introduce cytotoxicity to mouse cells, indicating its suitability as a medical implant material.

Researchers have been investigating microbial infections induced by medical implants and surgical instruments, which could lead to serious health complications^1^,^2^,^3^,^4^. Gram-positive *staphylococcus aureus* is one of the most common strains of *staphylococcus* and it is a pathogen capable of causing life-threatening infections (e.g., soft tissue infections), eye infections, pneumonia, meningitis, or even necrotizing fasciitis, and food poisoning^5^,^6^,^7^,^8^,^9^,^10^. Previous studies have suggested that copper ion (Cu^2+^) can serve as an antimicrobial agent^11^,^12^. Many antimicrobial copper alloys have been approved for use in many areas such as mass transit, medical facilities and buildings in the U.S.^13^.

Combining the merits of both structural and functional materials, antibacterial stainless steel (SS) is a new class of implant materials with broad-spectrum and long-term antibacterial effects. The precipitation of the copper-rich phases on the surface of cu-bearing SS and the Cu^2+^ released from the surface endow this novel material with broad-spectrum antimicrobial activities^14^,^15^,^16^,^17^. Some researchers demonstrated that copper kills bacteria by damaging their cell walls and cell membranes^11^,^18^,^19^. Cu^2+^ possesses strong reduction ability, it can adsorb electrons from bacteria, leading to cell lysis and oxidization of cell nuclei^20^,^21^. The reactive oxygen species (ROS) such as superoxide anions were generated by the catalysis of copper, resulting in the fatal damage to important cell constituents and DNA. The exact mechanisms to explain how copper kills bacteria are still under investigation^18^,^22^.

Although bacteria may be in the viable but non-culturable (VBNC) state, they can maintain their pathogenicity, and may become infectious when the environment improves^23^. Previous works indicated that frozen at a low temperature or direct culture following exposure to Cu^2+^ could induce bacteria into the VBNC state^24^,^25^. To prevent missing VBNC cells in cell counting, the live/dead cell staining method was introduced for viability and morphological determinations in addition to cell enumeration using the quantitative polymerase chain reaction (qPCR) method^26^,^27^,^28^,^29^. The cytotoxicity of the new material was necessarily evaluated^30^.

The aim of this study was to characterize a potential implant material with a good antibacterial ability and excellent cytotoxicity. In this study, both antibacterial performance and the cytocompatibility of the 317L-Cu SS after antibacterial heat treatment were investigated. The concentration of Cu^2+^ released from the 317L-Cu SS was also assessed.

## Materials and Methods

### Bacterial strain, culture medium and SS coupons

The Guide for Chinese Animal Care and Use Committee standards was followed for the animal housing and surgical procedures. All procedures were done in accordance with protocols approved by the Animal Ethics Committee of Chongqing University. The ubiquitous pathogen *S. aureus* (ATCC 25923) was used in this study. It was routinely subcultured and maintained at 37 °C in the Luria–Bertani (LB) medium (Booute Biotechnology Ltd., Wuhan, Hubei, China) before use. The MC3T3-E1 cell was cultured at 37 °C in the α-minimal essential medium (α-MEM) containing 10% (v/v) fetal bovine serum (Sigma-Aldrich Inc., St. Louis, MO, USA), 50 μg ml^−1^ gentamicin (Gen-view Scientific Inc., Galveston, TX, USA), 50 μg ml^−1^ ascorbic acid (Sigma-Aldrich Inc., St. Louis, MO, USA) and 2.5 μg ml^−1^ fungizone (Gen-view Scientific Inc., Galveston, TX, USA) in a Heracell 150i thermostatic incubator (Thermo Fisher Scientific Inc., Waltham, MA, US) containing 5% (v/v) CO_2_. The SS alloys used in this study included 317L-Cu SS (composition in Table 1) and commercial medical grade 317L SS (control). The 317L-Cu SS sample was heat treated under the following conditions: a solution treatment at 1100 °C for 0.5 h to distribute Cu evenly in the SS followed by water quenching, and then aging at 700 °C for 6 h in order to obtain the Cu-rich phases in the alloys^31^. The 317L SS control sample was treated in the same fashion. The heat treated alloys were machined to coin-shaped coupons for testing with a 10 mm diameter and a 1.5 mm thickness. Each coupon was degreased and cleaned by vortexing for 30 s in 10 ml acetone^32^.

### Atomic Absorption Spectroscopy (AAS)

The release of Cu^2+^ of 317L-Cu SS into a 0.9% (w/v) NaCl solution was measured using an atomic absorption spectrophotometer (Model, Z-2000; Hitachi Ltd., Tokyo, Japan). The detection wavelength and integration time for Cu^2+^ was 324.8 nm and 5 s, respectively. Five coupons were tested for each time point.

### The preparation for antibacterial test

Both 317L and 317L-Cu coupons were placed in a 24-multiwell culture plate. One ml *S. aureus* suspension with a concentration of 10^6^ CFU ml^−1^ was deposited into each well containing a coupon. The inoculated plate was incubated in a DNP 9272 incubator (Jinghong Corporation, Shanghai, China) at 37 °C for different periods (0.5 d, 1 d, 3 d and 5 d). All the antibacterial tests were performed under the relative humidity of 90% at 37 °C.

### Plate count method

At the end of each test time period, bacterial suspensions from the 24-well plates were diluted to approximately 10^3^ CFU ml^−1^, and 0.1 ml of the diluted bacterial suspension was streaked on an agar plate (Aoboxing Bio-tech Corporation, Beijing, China). The plates were then incubated at 37 °C for 1 d before counting the bacterial colonies^20^,^33^. The antibacterial rate C was calculated based on the following equation:


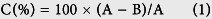


where *A* is number of CFUs for 317L SS and *B* for 317L-Cu SS^34^,^35^,^36^.

### Live/dead staining

To elucidate the antibacterial efficacy of 317L-Cu, *S. aureus* cells after incubation with 317L and 317L-Cu SS coupons were stained with the Live/Dead BacLight^TM^ kit (SYTO9 and propidium iodide (PI); Thermo Fisher Scientific Inc., Waltham, MA, USA). After incubation, a mixture of 10 μmol l^−1^ SYTO9 and 60 μmol l^−1^ PI was added to each well. To allow the diffusion of fluorochromes in the biofilm, the specimens were kept stationary in the absence of light at 37 °C for 20 min^37^. Then, the specimens were incubated for 30 min at room temperature in the dark. After gentle cleaning using 0.9% (w/v) NaCl followed by phosphate buffered saline (PBS) at pH 7.4, the coupons were retrieved for examination under confocal laser scanning microscopy (CLSM, Model C2si+; Nikon Ltd., Tokyo, Japan) equipped with a 40× objective lens with filters appropriate for SYTO9 and PI. Each coupon was mounted on a glass slab for CLSM imaging. The biofilm thickness was obtained using the 3-D scanning mode. The confluent areas with biofilm were randomly measured and 10 views for each sample were recorded for reporting^38^. All the biofilm thickness data were analyzed with the Nis-Elements Viewer version 4.20 software (Nikon Ltd., Tokyo, Japan)^21^. The number of sessile cells was quantified from the average of 10 sets of CLSM images using the ImageJ software (National Institutes of Health, Bethesda, MD, USA)^39^.

### DNA extraction and qPCR

Sterile coupons of both 317L SS and 317L-Cu SS were inoculated with 1 ml *S. aureus* suspension (10^6^ CFU ml^−1^). After incubation, bacterial suspensions were transferred to 5 ml sterile disposable centrifuge tubes with the DNase I (Takara Bio Inc., Dalian, Liaoning, China) treatment^40^,^41^,^42^. To collect the sessile cells, the coupons were transferred to 15 ml sterile disposable centrifuge tubes containing 10 ml sterile 0.9% NaCl with 20 mmol l^−1^ EDTA (to chelate free Cu^2+^) with 2-mm diameter glass beads and vortexed for 30 s. After that, both planktonic and sessile cells were mixed and pelleted by centrifugation. DNA from the pelleted cells was isolated and purified using the Takara Minibest Bacterial Genomic DNA Extraction kit (Version 3.0; Takara Bio Inc., Dalian, Liaoning, China) following the instructions for gram-positive bacteria. The purified DNA was stored at 4 °C before use.

The gene copy numbers of samples were quantified with the qPCR analysis using SYBR^®^ Premix Ex Taq^TM^ (Takara Bio Inc., Dalian, Liaoning, China) following the manufacturer’s instructions^43^,^44^. The qPCR to quantify *S. aureus* was performed with the primers nuc-F (5′ CCT GAA GCA AGT GCA TTT ACG A 3′) and nuc-R (5′ CTT TAG CCA AGC CTT GAC GAA CT 3′) targeted heat-stable nuclease^45^. The results were analyzed using the 2^−ΔΔCT^ method^46^.

### Transmission electron microscopy (TEM)

The *S. aureus* sessile cell membrane damage due to the biocidal action of 317L-Cu SS was investigated using TEM (Model H-7650; Hitachi Ltd., Tokyo, Japan) with an accelerating voltage of 80 kV. After incubation, the sessile cells were washed off from coupon surfaces with deionized water followed by 15 s of ultrasonication. The sessile cells were collected after centrifugation (6000 g, 5 min at 4 °C). The supernatant was discarded and deionized water was added to resuspend the sessile cells. Then the cells were fixed using osmic acid, dehydrated and placed on a copper grid for TEM observation.

### Scanning electron microscopy (SEM)

The surface morphologies of biofilms on 317L SS and 317L-Cu SS coupons were observed under SEM (Model S-3400N; CARL ZEISS Ltd., Oberkochen, BW, Germany) at operating voltage of 20 kV. After incubation, coupons were retrieved and washed three times using 3 ml 0.9% NaCl before being fixed with 2.5% (v/v) glutaraldehyde. Coupons were dehydrated with a graded ethanol series of 50%, 75%, 95% and 100% (v/v)^47^. The coupons were sputter coated with gold using the JFC-1200 Fine Coater (Jeal Corporation, Tokyo, Japan) before SEM imaging^48^.

### RTCA cytotoxicity assay

Each well was seeded with osteoblast-like 1,000 MC3T3-E1 mouse cells on a 96-well electronic plate (E-Plate View 96; ACEA Biosciences Inc., San Diego, CA, USA)^49^,^50^,^51^. The E-plate was read using the xCELLigence DP system (ACEA Biosciences Inc., San Diego, CA, USA) at 5-min intervals. The value of cellular index (CI) represents the morphological changes and the number of cells which can reflect negative effects on cellular functions. After 20 h of RTCA profiling, the assay was paused, and the plate was removed from the xCELLigence system. The liquid in each well was decanted and replaced with a 0.9% NaCl solution labeled as soaking solution that was exposed to 317L or 317L-Cu for a fixed time period (1 d, 3 d, or 5 d). The plate was monitored for 120 h. For each sample, there were two duplicates. Results were collected using the RTCA software (Version 2.0; ACEA Biosciences Inc., San Diego, CA, USA). The data expressed in CI units were exported to Microsoft Excel software (Microsoft Office Professional Plus 2010; Microsoft, Redmond, WA, USA) for mathematical analysis. The data were normalized to a starting CI of 1.0 at the time point immediately prior to the solution switch and addition of dices^52^.

### Cell morphological analysis

After 140 h of RTCA monitoring, the assay was terminated. A visual assessment of each treatment group was made, and photomicrographs were captured using a digital camera (Digital Sight DS-U3; Nikon Ltd., Tokyo, Japan).

### Statistical analysis

Statistical analysis was performed with SPSS 17.0 software. The transition of the number of bacteria between each coupon (by plate count, fluorescence staining and qPCR) and RTCA assay (by NCI) were analyzed by analysis of variance (ANOVA).

## Results

### Copper ion release curve over time

**Figure 1:**
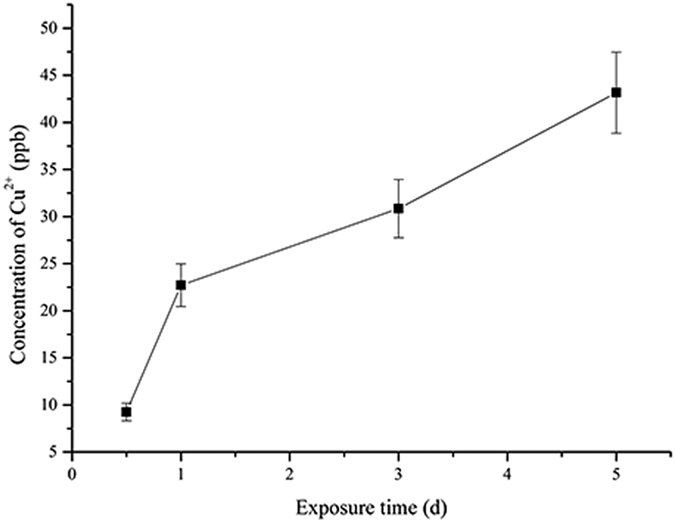
Cu^2+^ release in 0.9% NaCl by 317L-Cu SS over time.

The Cu^2+^ released from 317L-Cu SS into the 0.9% NaCl solution was determined using AAS. Figure 1 shows an increased concentration over time with a metal surface area to solution volume ratio of 78.5 m^2^ m^−3^. The daily release of Cu^2+^ from the 317L-Cu matrix was less than 20 ppb cm^−2^. This low level would not pose any immediate danger to human health^53^,^54^.

### Antibacterial efficacy of 317L-Cu SS

**Figure 2 f2:**
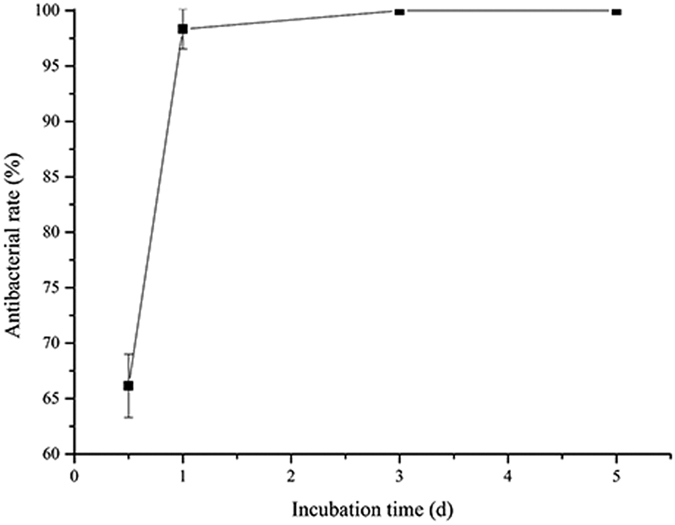
The antibacterial efficacy of 317L-Cu SS against *S. aureus* after incubation at 37 °C for 0.5 d, 1 d, 3 d and 5 d.

**Figure 3 f3:**
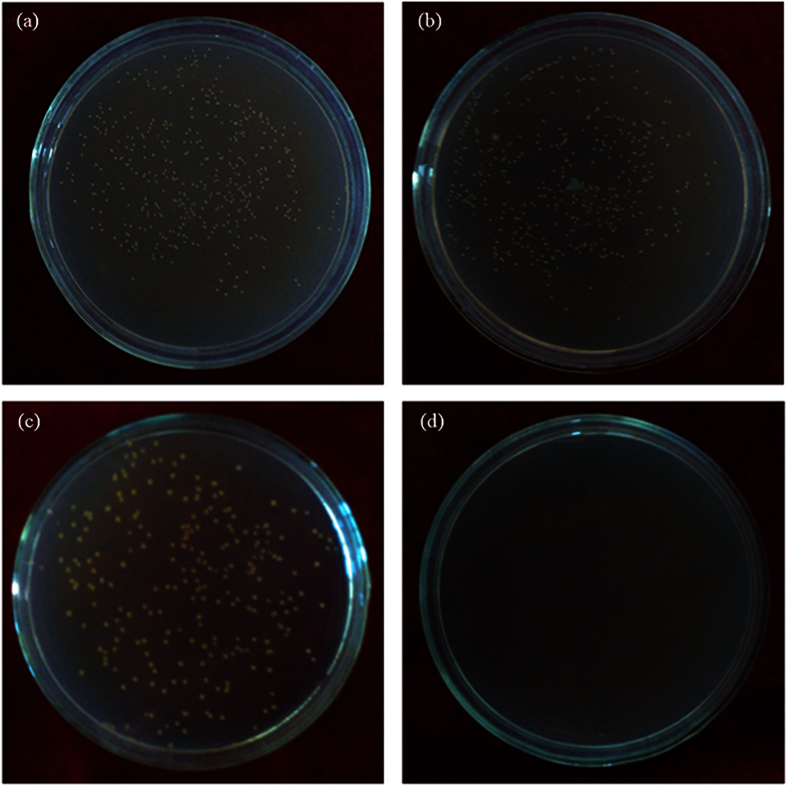
*S. aureus* colonies on agar plates inoculated with cell suspensions obtained from *S. aureus* liquid cultures exposed to 317L SS (**a**,**b**) and 317L-Cu SS (**c**,**d**) for 0.5 d (**a**,**c**) and 1 d (**b**,**d**), respectively.

Figure 2 indicates that the antibacterial rate of 317L-Cu SS reached up to 98.3% when the incubation time reached 1 d, which was consistent with the previously reported results^33^. The evaluation is considered as an effectively, widely used method. However, as mentioned, the results of this experiment can only determine that the 317L-Cu SS may inhibit the bacterial growth. Obviously, simply using the plate count method cannot guarantee the safety of a potential implant material. Figure 3 presents the plate cultures seeded with the cell suspensions obtained after *S. aureus* was incubated with the 317L SS and 317L-Cu SS coupons for 0.5 d and 1 d. It was found that after incubation with 317L-Cu SS for 1 d, no cells were detectable on nutrient agar plates. The 3 d and 5 d results (not shown) were identical to Fig. 3d, which confirmed that the good antibacterial performance of 317L-Cu SS showed after 1 d in 0.9% NaCl solution.

### The inhibition of *S. aureus* biofilm by 317L-Cu SS

**Figure 4:**
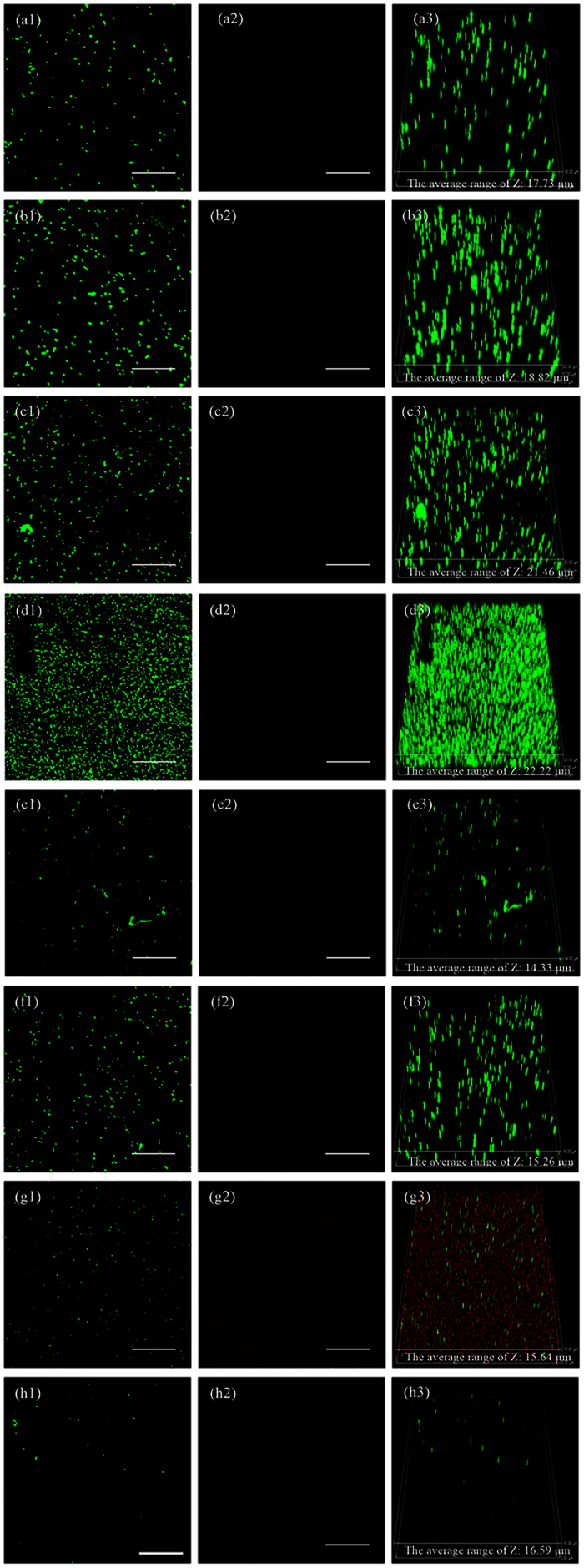
CLSM images of the *S. aureus* biofilm on 317L SS after 0.5 d (a1–a3), 1 d (b1–b3), 3 d (c1–c3) and 5 d (d1–d3) of incubation, and on 317L-Cu SS after 0.5 d (e1–e3), 1 d (f1–f3), 3 d (g1–g3) and 5 d (h1–h3) of incubation. (Left column for stained live cells only, middle column stained dead cells only and the right column for both). (Scale bar = 50 μm).

**Figure 5:**
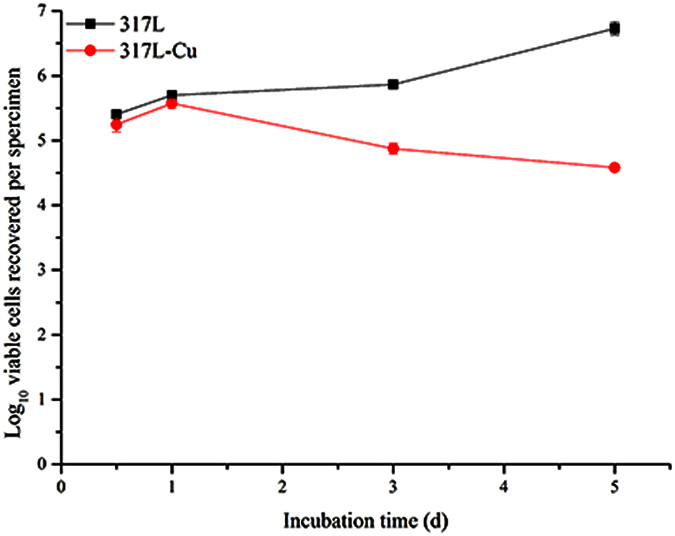
The number of viable bacterial cells stained with SYTO9 vs. time.

**Figure 6:**
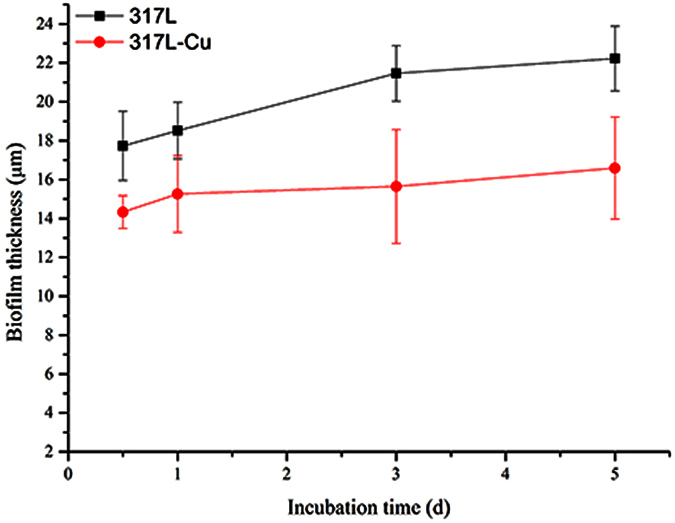
*S. aureus* biofilm thickness on a coupon vs. time.

Figure 4 shows the epifluorescent images obtained by CLSM showing viable (live) cells with integral membranes as green dots and non-viable (dead) cells with disrupted membranes as red dots. Viable cells were observed in the left column of each row while non-viable cells were observed in the middle column of each row. Viable and non-viable cells were observed simultaneously in 3D mode with the biofilm thickness of each coupon in the right column. In the initial 3 d, there were seldom dead cells on the 317L SS coupon surface, while more dead cells appeared on the 317L-Cu coupon surface after 1 d, indicating the satisfactory biocidal effect of 317L-Cu SS. For 317L-Cu SS, the proportion of non-viable cells increased significantly after 1 d, which corresponded to the Cu^2+^ release curve in Fig. 1. The result of Fig. 5 also supports that conclusion. The presence of non-viable cells on 317L SS was observed after 5 d, which might be caused by the normal cell death. Compared with 317L SS, the total number of viable and non-viable cells was obviously less on the 317L-Cu coupon surface, demonstrating that with the continuous released of Cu^2+^, the 317-L Cu possessed a satisfactory biocidal ability. The biofilm thickness values of *S. aureus* on 317L and 317L-Cu SS surfaces Fig. 6, show significant differences between 317L SS and 317L-Cu SS in each time point (P < 0.05).

### qPCR to quantify the antibacterial efficacy of 317L-Cu SS

The VBNC state can be summarized as a stationary state of microbial life, waiting for a suitable growth environment^55^. The plate counting method could not distinguish between dead cells and cells in the VBNC state. To verify that cells were killed by 317L-Cu SS rather than in the VBNC state, qPCR was used to evaluate the antibacterial efficacy of 317L-Cu.

**Figure 7:**
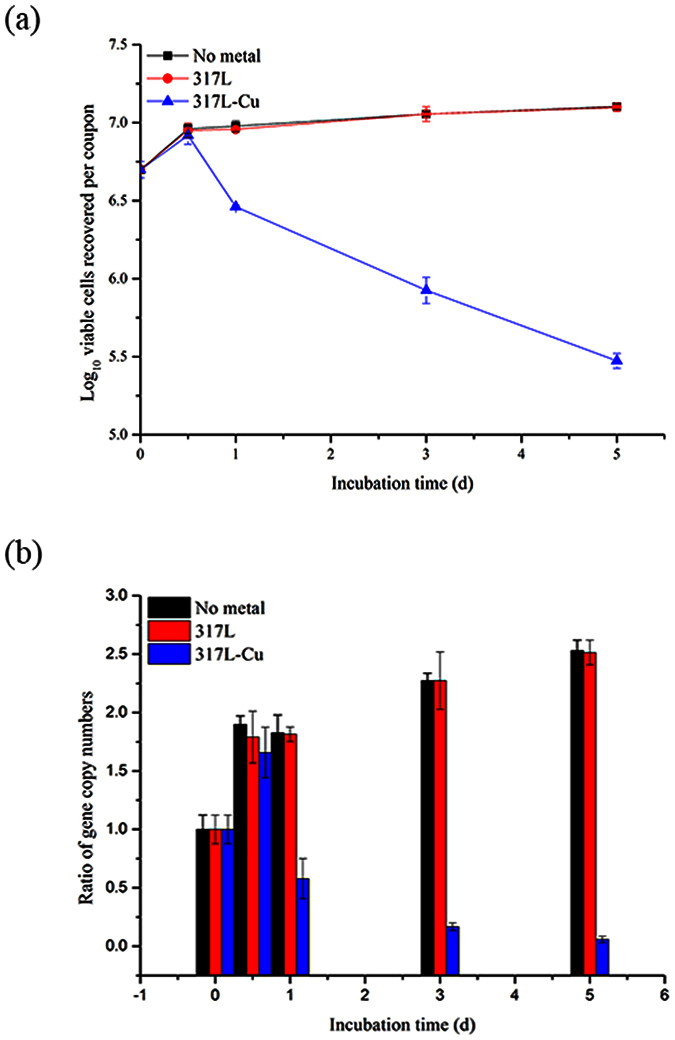
Calculated ratio of gene copy numbers on each coupon (mean values and standard deviations based on triplicate assays within a single qPCR setup): (**a**) The viable cells recovered and (**b**) bar chart version of (**a**) using the gene copy number before incubation as basis.

Figure 7a shows there were no orders of magnitude difference in viable cells recovered from 317L and 317L-Cu samples before 1 d. In conjunction with Fig. 1, the Cu^2+^ concentration was 9.25 ppb, probably insufficient to kill the sessile cells. Unlike pure copper, 317L-Cu took 3 d to release adequate Cu^2+^ to kill the sessile cells^21^,^32^. To visualize the data in Fig. 7a better, Fig. 7b was obtained by using the gene copy number before incubation as the basis. As seen in Fig. 7b, the reductions of genomic units (GU) by 2 orders of magnitude after 3 d and 5 d incubation with 317L-Cu SS were achieved. The trend in Fig. 7a is consistent with the results of Live/dead staining analysis shown in Fig. 5. In conjunction with the rationale behind Live/Dead staining and qPCR^56^,^57^, it was concluded that the Cu^2+^ not only led to cell membrane disruption but also gene reduction, confirming the biocidal efficacy of 317L-Cu SS.

### TEM and SEM to reveal the cell death process

**Figure 8:**
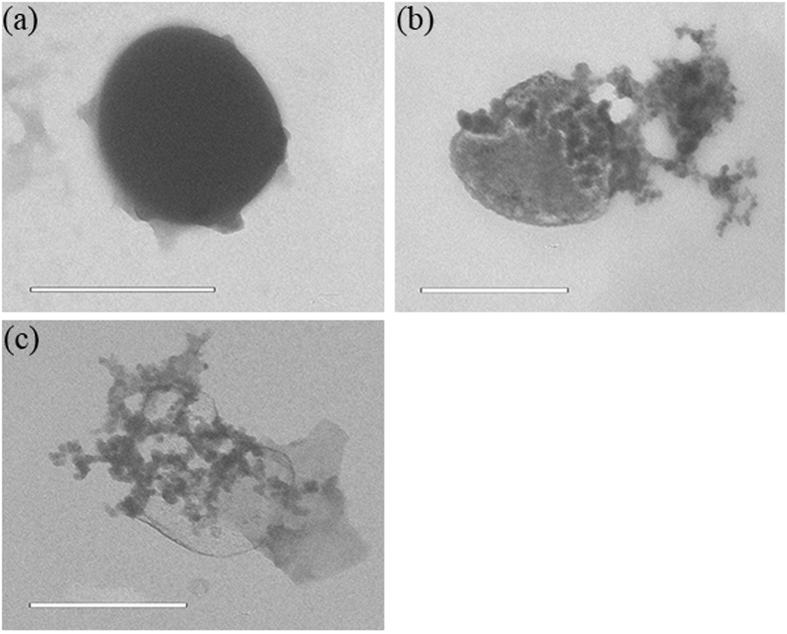
TEM images showing the deformation process of *S. aureus*: (**a**) well defined cells incubated with 317L-Cu SS after 0.5 d, (**b**) beginning of cell lysis of a cell incubated with 317L-Cu SS after 1 d, and (**c**) whole cell disintegration incubated with 317L-Cu SS after 3 d. (Scale bar = 0.5 μm).

The *S. aureus* ultrastructure influenced by 317L-Cu SS was assessed by TEM (Fig. 8). The healthy cells were shaped with the unaltered cell structure with the inner and the intact outer curved membrane. The consistent appearance observed in the control group (incubated with 317L SS) shows that *S. aureus* cells remained undamaged even after 5 d (Fig. 8a)^58^. When incubated with 317L-Cu SS, partial and complete destructions of cell membranes after 1 d were observed (Fig. 8b,c). These TEM images visually supported the 317L-Cu SS antibacterial ability against *S. aureus* by destroying its membrane.

**Figure 9:**
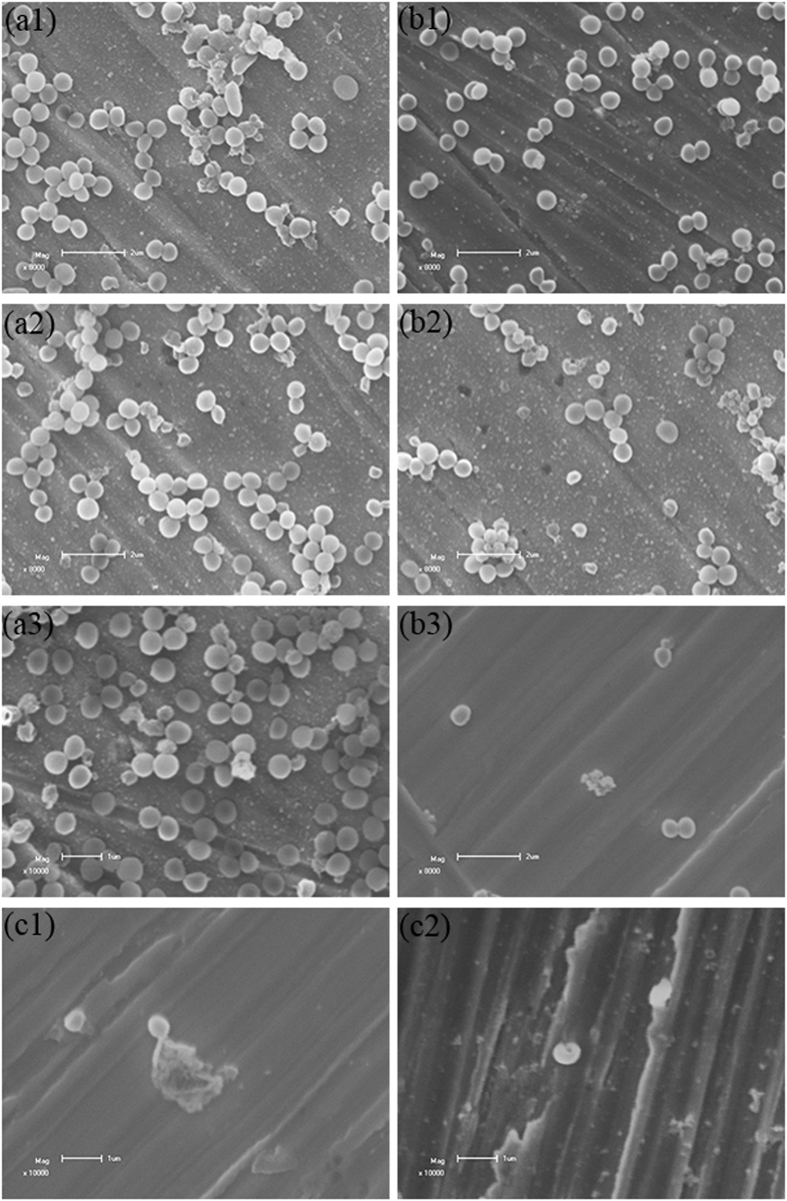
SEM images of *S. aureus* cells on coupons: (a1–a3) 317L SS after 1 d, 3 d and 5 d (scale bar = 1 μm), (b1–b3) 317L-Cu SS after 1 d, 3 d and 5 d (scale bar = 2 μm), and (c1–c2) observation of cell ultrastructure injury at 10000 × amplification for cells on 317L-Cu SS after 3 d (scale bar = 1 μm).

The surfaces of the 317L and 317L-Cu coupons were also investigated using SEM. Figure 9(a1–a3, [Fig f2], [Fig f3]) shows that after 1 d, 2 d and 3 d, there were no big differences of biofilm coverage on 317L. However, for 317L-Cu, the sessile cell coverage decreased after 3 d compared with 1 d. After 5 d, the sessile cells were much less. This trend was similar to that observed on Cu-bearing CoCrWNi alloys^36^. The cell integrity found on the 317L-Cu surface (Fig. 9c1–c2) was significantly damaged. This is consistent with the TEM result, indicating a satisfactory biocidal effect of the 317L-Cu SS.

### The cytotoxicity of 317L-Cu with mouse cells

**Figure 10:**
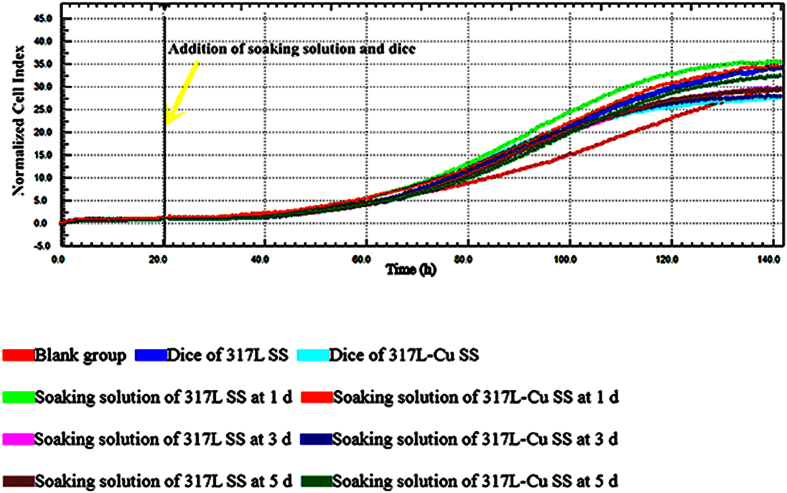
RTCA profiling of the soaking solution for mouse MC3T3-E1 cells with 1,000 MC3T3-E1 cells per well in α-MEM.

**Figure 11:**
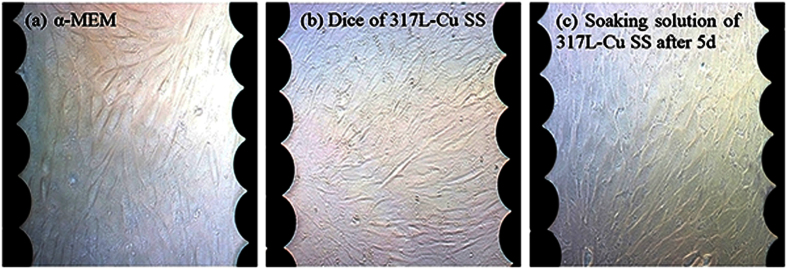
Representative photomicrographs of 24-well plates corresponding to 140 h in Fig. 10.

RTCA was carried out to compare 317L SS with 317L-Cu SS for their cytotoxicity to MC3T3-E1 mouse cells. A small cell concentration was required to prevent the quick exhaustion of nutrients prematurely. Therefore, based on the preliminary results, 1000 cells per well were seeded in each well on the E-Plate View 96, and the impedance was monitored every 5 min for 141 h. The characteristic RTCA growth profiles of MC3T3-E1 exposed to soaking solutions and SS dice are shown in Fig. 10. The morphological assessment under different treatments was shown in Fig. 11(a–c). At the end of the experiment, MC3T3-E1 grew healthily and there were no significant differences among the three groups^59^. Initially, the presence of the healthy cells led to an increase in the electrode impedance and the normalized cell index (NCI). When a soaking solution containing 317L and 317L-Cu dices was introduced, the cells might have a change in morphology, and could detach from the electrodes resulting in a change in NCI. In Fig. 10, both soaking solutions and dices did not lead to cell death (CI > 1) after 5 d. Figure 11 reveals a cell growth behavior which proliferated to form a full layer on the E-Plate View 96. No apoptosis cells were observed. The results here suggest that both types of SS did not introduce cytotoxicity to the mouse cells.

## Discussion

The JIS Z2801:2000 industrial standard based on plate count method was used to evaluate the antibacterial properties of antibacterial materials^60^. The defined antibacterial rate of 317L-Cu could reach up to 98.3% when the incubation time reached 1 d. Furthermore, based on the plate count method, the antibacterial rate was 99.99% at 3 d and 5 d, because there was no colony for the sample incubated with 317L-Cu after 3 d and 5 d. However, 317L-Cu SS was unable to kill the bacteria completely at 3 d and 5 d as indicated by qPCR. It was likely that the plate count approach was affected by the VBNC state of bacteria.

In order to obtain reliable results, live/dead staining have gained increased popularity among researchers in various fields of microbiology^29^,^61^. Researchers have used the JIS Z2801:2000 industrial standard in testing growth inhibition of bacteria, live/dead staining was used to determine the bactericidal effect^62^,^63^. In this procedure, live/dead staining depends on cytomembrane permeability to differentiate live and dead bacteria based on the assumption that the degradation of cellular DNA is incomplete^37^,^64^. Figure 5 shows that there was a 1–2 log reduction in viable cells after 5-d incubation with 317L-Cu SS and there was a significant difference (P < 0.05) between 317L-Cu SS and 317L SS, which was in agreement with previous reports that the cell membranes were the primary targets of contact killing through surface-released Cu^2+^ 11. Unfortunately, all staining procedures affect the structural integrity of the bacterial cells considerably^65^. Ethidium monoazide-quantitative PCR (EMA-qPCR) applies the same principles. It was found to be a poor indicator of cell viability, mainly because high concentrations of the dye can penetrate viable cells^66^. The qPCR with a prior DNase/PK treatment has been shown to be more practical in comparison with the PMA treatment^42^. It was able to eliminate the DNA from dead cells to obtain Fig. 7, which clearly proved the antibacterial efficacy of 317L-Cu SS. As mentioned above in the Fig. 3c discussion, there were no colony counts detectable on plates over 1 d. Combined with the analysis of qPCR, it was likely that most cells entered the VBNC state. However after 3 d and 5 d, Fig. 7 shows most bacterial cells were eradicated by the 317L-Cu SS^60^.

Researchers can directly observe cellular structures on solid surface after incubation using TEM and SEM^67^,^68^,^69^. To further investigate the mechanism of inhibition of Cu^2+^ on microorganisms, *S. aureus* was incubated with 317L-Cu SS and studied using TEM and SEM. Figures 8 and 9 clearly show that morphological changes in the injury and death of *S. aureus* cells after intubation with 317L-Cu SS.

Copper is an essential element but it is cytotoxic at elevated concentrations^19^. Based on the development of advanced sensor detection technology and cell culture technology, RTCA has been developed for *in vitro* cytotoxicity tests with reliable data outcome^70^,^71^. Here, E-plate was used because it was ideal for simultaneous visual and real-time analysis since these plates provided a viewing window for graphical analysis and electrochemical cytotoxicity test^52^. Compared with surface modification methods of implants, 317L-Cu SS can be regarded as an approach from the material aspect, which required no addition of the other antibacterial substances^72^,^73^. Not only 317L-Cu SS has long-lasting antibacterial effect, but also avoids the failure caused by surface wear or low adhesive force of coating^74^,^75^.

The preliminary study for a new type of antimicrobial metal was performed to observe its antibacterial performance, to investigate its antibacterial mechanism and to evaluate its biocompatibility. RTCA has been proved to have the characteristics of high sensitivity, high speed responsibility, real-time dynamic monitoring, label-free analysis^76^,^77^. Compared with conventional cell survival assay, RTCA can obtain more comprehensive and reliable data^78^. Unlike these cited studies, 5 d instead of 1 d was adopted as the incubation time to acquire the preliminary analysis of 317L-Cu cumulative toxicity. The results of three antimicrobial assessments and RTCA confirmed that 317L-Cu SS possessed a satisfactory antibacterial ability against *S. aureus* after 1 d. SEM and TEM images provided visual confirmations. Although the antimicrobial mechanism of Cu^2+^ is not fully established in the literature, this work provided additional evidence that Cu^2+^ led to cell membranes breakage and subsequent cell lysis. 317L-Cu SS possesses strong potential in medical applications to provide a metal surface that is easier to maintain a sanitary surface without antibacterial coatings or biocides. It is potentially a new biocompatible implant material with a good balance of long-term antibacterial ability and mechanical properties.

## Additional Information

**How to cite this article**: Sun, D. *et al*. An investigation of the antibacterial ability and cytotoxicity of a novel cu-bearing 317L stainless steel. *Sci. Rep.* **6**, 29244; doi: 10.1038/srep29244 (2016).

## Figures and Tables

**Table 1:** Chemical composition of 317L- Cu SS (wt%).

Element	Ni	Cr	Mo	Cu	Fe
317L-Cu	15.15	18.25	3.72	4.46	Balance
